# Correction: Can gender inequality be created without inter-group discrimination?

**DOI:** 10.1371/journal.pone.0244926

**Published:** 2020-12-31

**Authors:** Sylvie Huet, Floriana Gargiulo, Felicia Pratto

The Funding statement is incorrect. The correct Funding statement is as follows: This work was supported by the French National Agency, project ToRealSim (2019–2022) in the form of a grant awarded to SH (ANR-18-ORAR-0003).

## References

[1] Huet S, Gargiulo F, Pratto F (2020) Can gender inequality be created without inter-group discrimination? PLoS ONE 15(8): e0236840 10.1371/journal.pone.0236840 32780742 PMC7418958

